# Genome Database of the Latvian Population (LGDB): Design, Goals, and Primary Results

**DOI:** 10.2188/jea.JE20170079

**Published:** 2018-08-05

**Authors:** Vita Rovite, Yael Wolff-Sagi, Linda Zaharenko, Liene Nikitina-Zake, Elmars Grens, Janis Klovins

**Affiliations:** 1 Latvian Biomedical Research and Study Centre, Riga, Latvia; 2 National Program for Quality Indicators in Community Healthcare Braun School of Public Health & Community Medicine Faculty, The Hebrew University of Jerusalem, Israel

**Keywords:** genetic database, biobank, Eastern Europe

## Abstract

**Background:**

The Genome Database of the Latvian Population (LGDB) is a national biobank that collects, maintains, and processes health information, data, and biospecimens collected from representatives of the Latvian population. These specimens serve as a foundation for epidemiological research and prophylactic and therapeutic purposes.

**Methods:**

Participant recruitment and biomaterial and data processing were performed according to specifically designed standard protocols, taking into consideration international quality requirements. Legal and ethical aspects, including broad informed consent and personal data protection, were applied according to legal norms of the Republic of Latvia.

**Results:**

Since its start in 2006, the LGDB is comprised of biosamples and associated phenotypic and clinical information from over 31,504 participants, constituting approximately 1.5% of the Latvian population. The LGDB represents a mixed-design biobank and includes participants from the general population as well as disease-based cohorts. The standard set of biosamples stored in the LGDB consists of DNA, plasma, serum, and white blood cells; in some cohorts, these samples are complemented by cancer biopsies and microbiome and urine samples. The LGDB acts as a core structure for the Latvian Biomedical Research and Study Centre (BMC), representing the national node of Latvia in Biobanking and BioMolecular resources Research Infrastructure – European Research Infrastructure Consortium (BBMRI-ERIC).

**Conclusions:**

The development of the LGDB has enabled resources for biomedical research and promoted genetic testing in Latvia. Further challenges of the LGDB are the enrichment and harmonization of collected biosamples and data, the follow-up of selected participant groups, and continued networking and participation in collaboration projects.

## INTRODUCTION

Prior to the establishment of the Genome Database of the Latvian Population (LGDB), only a limited number of epidemiological studies evaluating risk factors that affect health and disease in the Latvian population were conducted.^1^^–^^4^ The main problems within the health related research fields included: limited number of available samples without standardized sample collection approaches, a lack of electronic medical records in the public health system, and non-computerized national disease registries. Additionally, the field of medical genetics in Latvia suffers from low genetic testing availability.

The initiation of the LGDB at a political level resulted from recognizing the power of population-based approaches to search for complex disease susceptibility genes, along with the need to develop the infrastructure for genetic testing. This would consequently serve in the public’s best interests, both in the short (ie, use in genetic testing) and the long run (research).

The establishment of the LGDB started with a scientific consultation process. Conducted by the Latvian Academy of Science and accompanied by media coverage, the process ensured transparency towards the public. Established under “The Human Genome Research Act”, LGDB operations were passed by the Latvian parliament in June 2002. The act became effective in January 2004, thereby ensuring the ethical, regulatory, and legal framework for the establishment and maintenance of the genome database.^5^ The Central Medical Ethics Committee of Latvia (CMEC) was appointed as the official overseeing body of the LGDB. Responsibilities of the CMEC included approving biobank protocols related to recruitment procedures.

**Figure 1.**
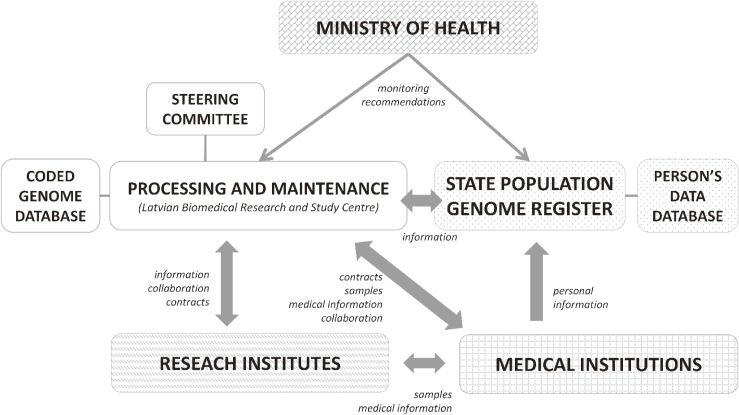
Structure of Genome Database of Latvian Population functioning

The Ministry of Health of the Republic of Latvia allocated funds, and the Latvian Biomedical Research and Study Centre (BMC) was consequently appointed as the main project processor on November 1, 2006. The State Genome Registry was established in order to manage participant’s associated personal data. A detailed plan of the LGDB governance is presented in Figure 1. The LGDB is maintained by the Genome Centre, which represents the core facility of the Latvian Biomedical Research and Study Centre. In the light of budgetary constraints the LGDB needs to strike a balance between sample size and the volume of generated phenotypic data. The aim of the LGDB development and functioning is to provide biological material, associated data, and infrastructure resource for the genetic and biomolecular research, disease prevention and health care evaluation at the national level. To achieve this objective, the main principles in setting the biobank were to develop and maintain the system for biosample and data processing and generate collections consisting of disease-specific cases and population-based controls for both retrospective and prospective epidemiological and biomolecular studies.

## MATERIALS AND METHODS

### Recruitment setting

Recruitment of participants started in 2003 with a pilot phase (January 2003 to October 2006) and is ongoing (from October 2006) through the official establishment of the LGDB. The general inclusion criteria are the following: participants must be residents of Latvia, over 18 years old, and without intellectual disability. There are also specific provisions in Human Genome Research Law defining the requirements for the involvement of children under the age of 18 and people with intellectual disability. Recruitment is performed through the population-based setting, study- and disease-specific segments, and volunteers from the general population. Population-based recruitment is carried out by inviting randomized individuals from State registries, and people are invited to attend any of the recruitment centers located in numerous health care institutions (hospitals and specialty doctor’s or general practitioner’s offices). Study- and disease-specific recruitment is done by a medical doctor or a trained personnel through the existing LGDB recruitment centers (see below) or at the location specified by the individual study. Volunteer participants from the general population are invited to participate through the local media (newspapers and television interviews) or general practitioners. Upon contacting LGDB, volunteers are appointed to attend recruitment spots at health care centers.

Recruitment centers are established at various health care institutions, where the personnel is instructed about the general inclusion criteria, the project-specific inclusion criteria (depending on the study or disease group), and standard operation procedures (SOPs) for biological sampling and material handling; interviewers are also instructed about the administration of questionnaires and the collection of anthropometric measurements. A detailed list of the institutions that serve or have served as recruitment centers is presented in eMaterial 1.

### Consent, informative procedures, and confidentiality

All LGDB participants sign an informed consent form (approved by CMEC) after receiving a thorough explanation regarding the research project and database. Participants do not receive payment for their contribution. All of the procedures described below are defined under “The Human Genome Research Act” and those regulations set forth by the Cabinet of Ministers.

The informed consent form consists of text describing the aims of the LGDB, potential benefits and risks, confidentiality and legal frameworks, and a list of the following restrictions that participants may choose to apply to the use of their own samples and data: 1) permission to export the samples or data abroad, 2) permission to use the connected medical record information for research purposes by LGDB staff, and 3) any further restriction specified by participants upon investigation of their genomic data. The consent form allows the use of the participant’s data and material for different research purposes that do not require the participant to be contacted for project-specific basis (taking into account the restrictions specified in the form). This document is signed in duplicate—one for the participant and one that is stored at the State Genome Registry. The translation of the informed consent form is included in eMaterial 2.

Participants have the right to withdraw their consent. In such cases, all data and biosamples are destroyed according to defined procedures. Participants may also choose to receive feedback on their individual genetic results in the event of clinically significant findings. In this case, the State Genome Registry invites the participant to meet with a LGDB geneticist who communicates the results, and appoints a repeated analysis under a diagnostic setting.

The participant may also choose not to be informed of any individual results or those results relating to a specific, incurable condition caused by a genetic defect. Regardless of this choice, any participant of the LGDB may at any time, make an enquiry to obtain the full information stored in the LGDB.

LGDB operates at two distinct locations in order to enable an efficient privacy-protection process. The identity of participants is double coded. Consent forms and other collectable items and biospecimens obtained from newly recruited persons are supplied a “transportation code”. The transportation code includes information about the sample: study code, code of the doctor that recruited the participant, code of medical institution, code of interviewer, and other information regarding the sample. Upon arrival at the LGDB, the transportation codes are physically removed from the consent forms and replaced by 16-digit barcodes. Forms containing participants’ personal information (name, surname, persons’ ID, reported address) are submitted and stored both electronically and in the form of paper for a period of 75 years in the State Genome Register of the Latvian Population, which is maintained by the Centre for Disease Prevention and Control of the Ministry of Health. Participants’ biological samples and questionnaire data are stored at the BMC, solely coded by their unique 16-digit codes. Samples are decoded, and the link between a coded sample and a physical person is reestablished by the State Genome Register based on the request from the LGDB or the participant only in cases provided in the Human Genome Research Law: 1) to obtain additional health data or a biological sample; 2) to destroy samples and data upon a participant’s request; or 3) to provide research results to a participant or his/her doctor, and other cases provided by the law.

### Biosample collection and storage

Each LGDB participant contributes 20 mL of EDTA blood and 7 mL of blood in a clot-activator tube for serum separation. Serum, plasma, and white blood cells are separated within 2 days from the date of collection. DNA is extracted using the phenol extraction method (detailed protocol available in eMaterial 3). Plasma, serum, white blood cells, and DNA aliquots are stored at a temperature of −80°C. Throughout the process, a laboratory information and management system (LIMS)-controlled workflow is used, and includes 2-D barcoded tubes for sample identification.

A NanoDrop ND-1000 spectrometer (PeqLab, Erlangen, Germany) is used to measure the concentrations of extracted DNA, and a 260/280 OD ratio is used to estimate the quantity and purity of the product.^6^ The DNA sample is considered to be of good quality if the concentration is over 100 ng/mL and the OD ratio is between 1.8 and 2.0. Absence of DNA degradation is verified using agarose gel electrophoresis.

Upon request and for selected samples, Qubit™ fluorometric quantitation (Life Technologies, Thermo Fisher Scientific Inc., Waltham, MA, USA) is used for further quality control of the extracted DNA.

Biosamples stored in the LGDB comprise five aliquots of 1 mL plasma stored at −80°C, three aliquots of 1 mL sera stored at −80°C, one aliquot of 0.5 mL white blood cells stored at −80°C, and two aliquots of 1 mL DNA stored at −20°C.

Samples are available for research purposes. A special committee that considers sample request applications evaluates the research design, amount of sample required, rationale, and expenditure. Applicants must provide information relating to the approval of the proposed research by an ethics committee, which, if relevant, includes an evaluation of requests to ship samples abroad. Upon the approval of the application, the involved parties sign a material transfer agreement. This agreement stipulates the permitted actions toward samples, reporting of research results back to the LGDB and other activities concerning samples, associated information, and research results obtained.

### Collection of phenotypic and clinical information

The health and hereditary core questionnaire contains questions on personal disease history, medication intake, physical activity, nutrition, smoking habits, alcohol consumption, education level, marital status, age, gender, ethnicity, income, working status, and family history of diseases and longevity (a detailed list of the available data is presented in eMaterial 4). Using electronic systems, questionnaires are administered by trained interviewers using phone or face-to-face interview methods. Coded data from the health and hereditary questionnaire generated by the LGDB was included in the SAIL data linking and harmonization project, as described by Spjuth et al.^7^ LGDB data currently eligible for phenotype searches and analysis in SAIL includes that obtained from 3,769 participants. Using a separate form, medical diagnoses are confirmed by physicians using International Classification of Diseases (ICD)-10 codes.

The type and mode of data collection for anthropometric and biochemical parameters differ depending on the involvement of the scheme. Nurses generally obtain anthropometric parameters at the time that participants are recruited at medical facilities. The participant’s weight is measured barefoot and in light clothing, and is recorded in kilograms to the nearest decimal value. Height is measured barefoot and is recorded in centimeters. Two non-consecutive blood pressure measurements are taken—one in the very beginning of the session, and one at the end of it. In some recruitment schemes, self-reported height and weight are obtained.

The information about participants’ life style and dietary habits is obtained using questionnaires that are based on examples in literature; included questions assess relevant data of a person’s life history, current status, and other important factors, such as smoking, physical activities, and alcohol consumption. The dietary questionnaire is designed to give an overview of a person’s dietary habits regarding the type, amount, and frequency of food and liquid consumption. These questionnaires are available upon request.

Total cholesterol, low-density lipoprotein cholesterol, high-density lipoprotein cholesterol, and fasting glucose concentrations are obtained from participants that donate blood at biochemical laboratories. Results are reported, if stipulated within the framework of the research project, to the participant, the recruiting physician(s), and to the LGDB. Additionally, biochemical tests that are conducted on stored LGDB plasma samples are achieved using RXimola technology (Randox Laboratories, Belfast, United Kingdom). Use of this system, however, does depend upon availability of excess financial resources, with the goal of enriching biochemical data available for stored biosamples. All information on blood biochemical tests is stored electronically in the LGDB database, with a detailed list of biochemical test results that have been collected shown in eMaterial 5.

### Data architecture

For sample and data administration, Nautilus-based LIMS (Delphi IT Technology, Boston, MA, USA) is used. Each patient has a record that is stored in an Oracle database (Oracle corporation, Redwood Shores, CA, USA). The LGDB stores the data regarding the samples with the 16-digit codes, there are separate parts of an Oracle database each devoted to specific data: phenotype, genotype, biosample information, biochemical test results, associated metadata, and other information. The link between the separate tables in the database can be established via the 16-digit code.

The Nautilus 8.01 LIMS system provides sample input, data tracking and management, label printing, and record exportation functions. Data are exported from the database using Microsoft Access.

### Genetic analysis

The majority of DNA samples supplied to the specific projects from the LGDB were tested in-house using facilities available at the Genome Centre. Genetic analysis methods used at this center include: TaqMan^®^ SNP Genotyping Assays (Life Technologies) on ViiA7 and 7500 Real Time PCR systems (Life Technologies), single nucleotide extension analysis on a MALDI-TOF mass spectrometer (BRUKER DALTONIK GmbH, Bremen, Germany), GoldenGate genotyping with VeraCode Technology (Illumina Inc., San Diego, CA, USA), Sanger sequencing on an ABI PRISM 3130xl genetic analyzer (Life Technologies), and massive parallel sequencing using Ion Torrent PGM and Ion Torrent Proton sequencing systems (Life Technologies).

Genotyping quality control is performed routinely using 1–10% within-run and between-run duplicates (sample-size dependent). Additionally, direct sequencing is performed in order to confirm any findings bearing diagnostic implications, as well as for the routine control of genotyping and Next Generation Sequencing (NGS) procedures. Mainly in the context of international genome-wide association studies (GWAS), GWAS genotyping has been performed on a limited number of samples.

## RESULTS

### Established collection

Prior to the establishment of the LGDB, a pilot sample collection study was initiated by a network of 15 Latvian research groups involved in the collaborative program known as the “Genomic studies of the Latvian population, their application for diagnosis and prevention of human pathology”. The pilot study was conducted under the leadership of the BMC between January 2003 and October 2006. During this period, the biobank infrastructure was developed according to the framework established by the Structural Fund project under the banner of the European Regional Development Fund Programme. Running between 2004 and 2006, 2,594 participants were recruited through disease-orientated projects.

This ongoing recruitment scheme is also voluntary, and recruitment is performed via communication with a specialty doctor. Disease-orientated projects represent the majority of the participants that have involved in the LGDB and employ variable recruitment strategies. These strategies include not only disease-based cohorts, but also respective control groups and random sampling units within specific hospitals or general practices. Currently, 85% of the LGDB participants have been recruited via study- or disease-based research, 12.1% through randomized population sampling, and 2.9% as volunteers from the general population. To date, these various recruitment strategies include 31,504 recruited participants. Table 1 lists the recruitment schemes, projects, and the number of participants included in each segment.

**Table 1.** Donor recruitment segments of Genome Database of Latvian population

Recruitment type	Recruitment site	Number of donors	Period
Research of various endocrinological disorders	Pauls Stradins Clinical University Hospital	4,682	2007–ongoing
Population based screening program	Various recruitment sites	4,236	2013–ongoing
State registry population based recruitment	Pauls Stradins Clinical University Hospital	3,807	2008–2009
Research of various cardiological disorders	Pauls Stradins Clinical University Hospital	3,476	2007–2014
Research of various oncologic disorders	Oncology centre of Latvia	2,798	2009–ongoing
Research of various gastrointestinal disorders	Pauls Stradins Clinical University Hospital and Riga East University Hospital	2,333	2008–ongoing
Coronary heart disease study	Pauls Stradins Clinical University Hospital	1,387	2003–2008
Breast and colorectal cancer study	Oncology centre of Latvia and Pauls Stradins Clinical University Hospital	884	2003–2008
Volunteer participants	Volunteering participation	727	2008–ongoing
Research of various venous disorders	Various phlebology clinics	533	2011–ongoing
Research of tuberculosis	Tuberculosis and lung disease centre	435	2014–ongoing
Lat-Diana - type 1 diabetes patients	Pauls Stradins Clinical University Hospital	404	2013–ongoing
Familial hypercholesterolemia study	Pauls Stradins Clinical University Hospital	399	2012–ongoing
OPTIMED – longitudinal cohort of type 2 diabetes patients	Various recruitment sites including hospitals, family doctors and endocrinology doctors	386	2010–ongoing
Population based healthy volunteer donors	Recruited at family doctors	277	2008–2009
MODY patients and family members	Various recruitment sites including hospitals, family doctors and endocrinology doctors	219	2011–ongoing
Research of melanoma	Oncology centre of Latvia	217	2004–2008
Research of various rheumatologic disorders	Pauls Stradins Clinical University Hospital	183	2008–ongoing
retro-OPTIMED - longitudinal cohort of type 2 diabetes patients	Various recruitment sites including hospitals, family doctors and endocrinology doctors	171	2011–2014
Chronic lung disease study	Pauls Stradins Clinical University Hospital	164	2010–2014
Grave’s disease study	Pauls Stradins Clinical University Hospital	146	2004–2008
Pituitary adenoma study	Pauls Stradins Clinical University Hospital and Riga East University Hospital	122	2006–ongoing
Essential tremor study	Pauls Stradins Clinical University Hospital	119	2010–2014
Atopic dermatitis study	Clinic of Dermatology Institute of Latvia	74	2007–2008
Pregnant women	Riga Maternity House	66	2014–ongoing
Different recruitment strategies recruited less than 50 donors each	Various recruitment sites including hospitals, family doctors and health care practitioners	—	2003–ongoing

The Latvian Population Registry served as the sampling frame for the population-based phase of the LGDB in a collaborative initiative performed by the Research Institute of Cardiology, University of Latvia. The stratified random sampling was performed for a total of 6,000 persons. Out of these, 63.5% (*n* = 3,807) agreed to participate and provided full information and biosamples. Detailed information regarding the sampling method, data collection, and population characteristics is given by Erglis et al.^8^

In total, 922 participants (2.9%) have voluntarily enrolled in the database from the general population, which represents the cohort for additional control samples and helps to increase the public awareness of health research significance.

### Population characteristics

**Figure 2.**
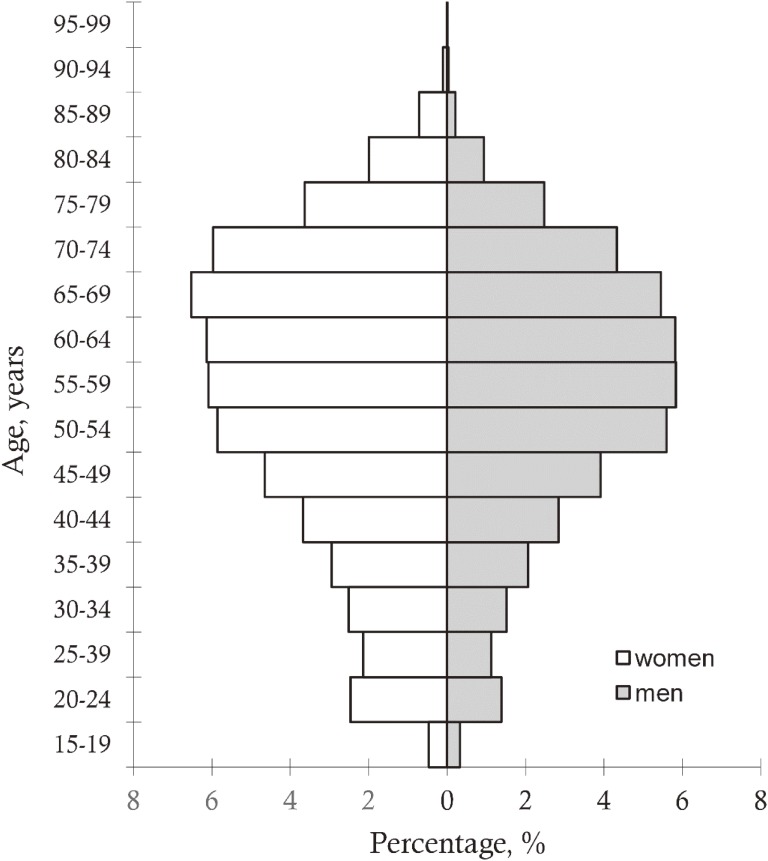
Age and sex distribution in Genome Database of Latvian population for period from 2003 to 2017

The study population consists of individuals in the age range of 18–99 years, with a mean age of 55.4 (standard deviation, 15.9) years and a median age of 58 years. Of those included in the database, 56% are female. The database mainly represents older adults, as 78.8% of the population fell within the age range of 40–79 years at the time of enrollment. The age and gender distribution is shown in Figure 2.

A question regarding self-reported nationality indicated that 60.8% of LGDB participants are Latvian, while 27.8% are Russian. The remaining nationalities include 3.9% Belarussian, 3.1% Ukrainian, 2.2% Polish, 1.3% Lithuanian, and 0.9% other. This distribution is highly conserved to that of ethnic group distribution in Latvia, as reported by the 2011 population census. This census indicated 62% Latvian and 27% Russian nationalities, while the remainder included 3.3% Belarussian, 2.2% Ukrainian, 2.2% Polish, 1.2% Lithuanian, and 2.1% other.^9^

Participants born in Latvia were also asked to report their place of birth, defined according to the five districts of the country. Again, the distribution was similar to that of the general population, with 32.1% coming from Riga, 20.8% from Vidzeme, 20.2% from Latgale, 14.1% from Kurzeme, and 12.8% from Zemgale. For the purpose of comparison, the 2011 Latvian census indicated the following residential distribution: 35% in Riga, 24% in Vidzeme, 15% in Latgale, 13% in Kurzeme, and 12% in Zemgale.^9^

In total, 5.2% of the study population were reportedly healthy or lacked a medical diagnosis of disease. It should be noted that the total number of ICD10 diagnoses exceeds the number of participants (*n* = 31,504), since many participants have more than one reported diagnosis (*n* = 47,015 diagnoses, in addition to the 1,612 individuals defined as healthy). The main medical conditions within the study populations essentially include hypertension, cardiovascular diseases, type II diabetes, and oncological conditions. Details regarding the frequency of diagnoses within this study population are given in Table 2.

**Table 2.** Frequency of diagnoses of Genome Database of Latvian population participants

ICD-10 Diagnoses groups	*N*
Cardiovascular (Ix)	17,747
Essential hypertension (I10)	5,473
Heart failure (I50)	3,173
Myocardial infarction (I21)	2,448
Angina pectoris (I20)	2,141
Chronic ischemic heart disease (I25)	1,843
Endocrine (Ex)	8,955
Type 2 diabetes mellitus (E11)	2,524
Dyslipidemia (E78)	2,240
Type 1 diabetes mellitus (E10)	814
Overweight and obesity (E66)	800
Oncology (Cx)	3,747
Breast cancer (C50)	796
Gastric cancer (C16)	675
Colon cancer (C18)	351
Prostate cancer (C61)	329
Melanoma (C43)	307
Ovarian cancer (C56)	194
Rectal cancer (C20)	178
Pancreatic cancer (C25)	151
Thyroid cancer (C73)	115
Cervical cancer (C53)	99
Lung cancer (C34)	89
Rectosigmoid junction cancer (C19)	64
Digestive system (Kx)	3,410
Musculosceletal and connective tissue (Mx)	1,674
Genitourinary system (Nx)	1,272
Nervous system (Gx)	1,254
Blood, Blood-forming organs, immune mechanisms (Dx)	1,149
Respiratory system (Jx)	853
Diseases of the eye and ear (Hx)	685
**Total *N* of diagnoses**	**47,560**
*Reported healthy/No diagnoses*	*1,612*

### Participation in research projects

The LGDB has been successfully serving as a research platform for numerous genetic and biomarker research projects since its establishment in 2006. A subset of 91 Latvian individuals were included in a phylogenetic study, which estimated and compared the genetic structure of different European population groups.^10^

Subsets of the LGDB have been included in consortia,^11^ collaborative GWAS projects,^12^^,^^13^ and replication and validation studies.^14^^–^^19^ However, the LGDB mainly serves as a platform for local research, where the main areas of interest currently include: autoimmune diseases, cardiovascular diseases, endocrine diseases, cancer-related biomarker research, studies on monogenic diseases, mitochondrial disorders and longevity, and pharmacogenetic studies. Detailed information regarding research studies that have been using LGDB resources are given in eMaterial 6.

Biobank-related research has examples of direct interaction with the Latvian health care system, such as in the case of the Celiac disease study or hemochromatosis.^16^^,^^20^ Thus, in the case of Celiac disease, the prevalence of the disease in the Latvian population was established by means of serology and genetic testing of 1,444 individuals from the population-based section of the LGDB. The prevalence of Celiac disease found in this study was less than 0.5%, and was lower than previous assumptions (1–1.5%). These results were further communicated to the general practitioners, and served to update the guidelines for diagnosis.^20^

To further study the prospective outcome of genetic effects the LGDB has performed pilot studies of follow-up of recruited oncology and type 2 diabetes patients by linking the date of death obtained from the State Death Registry to the research results. Additionally, cohorts of type 2 diabetes and gastrointestinal disease patients have been followed-up through the State Genome Registry to obtain and renew additional clinical information via visit to a specialty doctor. Results of these studies are still to be published. Recently, the legal regulations for the obtainment of data for the research from medical documentation and disease registries have come in to force in Latvia (Procedures for Using the Patient Data in a Specific Research Cabinet of Ministers Regulation No. 446), so additional follow-up and epidemiological studies could be made.

Since 2016, the Republic of Latvia has been approved for admission as a member of the BBMRI-ERIC (European Research Infrastructure Consortium), and Latvian Biomedical Research and Study Centre was appointed as National node.^21^ Participation in BBMRI-ERIC extends opportunities for participation in collaborative international research projects.

## DISCUSSION

The aim of the development of the LGDB was initially to promote research focused on human disease epidemiology, genetics, and biomedicine in Latvia. To date, numerous high-impact studies have been performed that would have not been possible without the use of LGDB samples. These research projects have used different assets of the LGDB, including biosamples, such as DNA, plasma and sera, and sample-associated phenotypes. However, there are topics to be considered for the further development and evolution of the LGDB. These topics, for example, include the quality of obtained biomaterial and preservation thereof, cost effective expansion of the biobank, cohort improvement, and clinical data availability, as well as networking with the biobanking community for research and data-sharing purposes.

One of the major concerns of the biobanking field is the assurance of preanalytic quality of preserved biomaterials. Many efforts have been made within biobanking networks to develop standards that would fulfill requirements of research communities. However, use of samples from several biobanks has brought up concerns regarding the different protocols used for sample processing and storage. This could affect the state of the sample and research results if these manipulations influence the parameters under investigation.^22^^,^^23^ The solution to this problem is the use of specific, standardized procedures and protocols. This would ensure that the samples collected and stored in different biobanks are processed in compatible ways. The LGDB sample processing method is designed to comply with OECD and IARC guidelines.^24^^,^^25^ However, new documents for molecular in vitro diagnostic examinations containing specifications for pre-examination processes (CEN standards) have been released. Compatibility of the LGDB procedures with these protocols will be crucial for ensuring sample quality for international collaboration projects.

The LGDB recognizes that long-term storage of samples in liquid nitrogen (below −160°C) instead of −80°C freezers would prolong biomaterial sustainability, as indicated in numerous literature resources.^26^^,^^27^ Other biobanks also store their plasma and sera samples at −80°C,^28^^,^^29^ as a large number of bioanalytes seem to be stable at this temperature.^30^ Biospecimens from the LGDB are currently comparable to samples from other biobanks and meet the standards proposed by other biocollections. There are also strategies to divide biocollections in working material archives (−80°C) and back up archives (−180°C).^28^ This facilitates the cost effectiveness of maintaining biobank specimens.

Another widely discussed point in the development of the LGDB is financial sustainability.^31^^–^^33^ The main financial strategies include the compilation of a business plan; strengths, weaknesses, opportunities, and threats analysis; cost effectiveness planning; improvement in sample quality; supply and demand evaluation; and the formation and expansion of collaboration with industries.^31^^–^^33^ From the initial establishment the basic LGDB, financial support was provided through national funds from the Ministry of Health of the Republic of Latvia. Research-based resources funded the development of some of the segments of the LGDB. The additional steps that have supported infrastructure development and maintenance include the introduction and approbation of specific genetic tests used for diagnostic purposes by commercial companies, and DNA isolation and genotyping services for the research community. Furthermore, it would be useful to explore possibilities of extending collaborations with pharmaceutical and medical industries for the provision of samples for research purposes, or conducting research studies under contractual agreements. Accordingly, it would be necessary to perform a careful cost evaluation for sample processing and maintenance in order to have transparent prices for the use of biomaterial and improved cost effectiveness and quality. It is indicated in literature that funds must be assessed for biobank exit plans as well.^32^

Presently, supply and demand models indicate that LGDB samples are requested for both disease-specific cases and control groups. These biomaterials are used for research-based studies. As mentioned previously, LGDB recruitment includes several strategies in both disease-orientated and population-based segments. Different recruitment strategies are likely to facilitate a varied array of biobank specimens and provide opportunities for larger sample maneuverability. There has been a high demand from the LGDB for control samples representing the general population in a wide range of studies. Therefore, especially with regard to sera and plasma material, expansion of this biobank segment is required. In the disease-orientated segment of the LGDB, more than 15,000 cardiovascular-related biomaterials, more than 7,000 endocrine-related biomaterials, and more than 3,000 oncological biomaterials are housed. Overall, these materials have sustained the supply required for the local research community. However, the LGDB recognizes the need of more focused sample collection and cohort development strategies in the future. Based on statistical evaluation, a biobank containing samples from 20,000 middle-aged recruits will generate 10,000 incident cases of common diseases like cardiovascular conditions and diabetes.^34^ Currently, the LGDB has a recruitment history of more than 10 years. This is sufficient to generate cohorts of previously recruited participants by the obtainment of novel biomaterials and phenotypic and clinical information. The development of clinically well-annotated samples and rare disease cases would enrich the LGDB collection. Such biomaterials have higher grading rates for use, thereby having more meaningful applications.^35^

The key factor in the development of the LGDB would certainly be sample size rationale. A major issue in the design of a national biobank and genetic database is recognizing the necessity of data harmonization and synthesis across multiple international studies in order to ensure sufficient statistical power of future analyses. The LGDB aims to acquire 60,000 study participants. However, this includes a varying number of disease-specific patients and control groups. When robustly taking into account the analytical complexity required (including assessment errors), the estimated sample sizes for analyses of main causal effects are typically in the thousands, while analyses involving gene-gene or gene-environment interactions would usually require tens of thousands of samples.^34^ This emphasizes the importance of having a platform that allows data pulling and meta-analyses. This necessity is further accentuated when considering studies that may reveal loci that exert a small effect on the studied phenotype, as well as the effects of rare genetic variations.^36^

For common diseases, sample sizes of 4,000 cases and 4,000 controls are required to reach the 80% statistical power needed in human genome epidemiology experiments. The statistical power could be improved by a case-control ratio of 1:4.^34^ In some specific conditions, like myocardial infarction and type II diabetes, the LGDB could meet the required need. However, in specific cancers or other diseases, research LGDB samples could be used in collaborative studies including biomaterials from several repositories in order to attain sample sizes that overcome statistical restraints.

As noted by Zawati et al, the legal and ethical frameworks of different population biobanks do not, in many instances, provide clear instructions in order to obtain international access.^37^ However, the defined mission of the LGDB is not only to create and maintain a national system of genomic and health information storage and data processing capabilities, but also to share genetic and phenotypic data with scientists in Latvia and abroad and to enhance collaborative research. This is reflected in the formulation of participants’ informed consent, where explicit permission is given (or denied, if a participant so chooses) to the LGDB for the “exportation of tissue samples and medical records for investigation abroad”.

Apart from the aspect of achieving sufficient statistical power, data sharing is increasingly being regarded as necessary from an ethical point of view. This is because it allows the contributions of participants to be respected to a greater extent^38^ and also reflects the view that scientific knowledge belongs to the general public.^37^ Hence, the LGDB was designed to be compatible to the Biobanking and Biomolecular Resources Research Infrastructure (BBMRI) framework and is included in the Public Population Projects in Genomics (P3G) repository.^39^

Currently, the LGDB is a fully functional biobank that continues activities involving the collection of biomaterials and associated phenotypical, clinically relevant information necessary for research purposes. The objectives for future developments involve the improvement of sample processing for quality purposes, data enrichment, and cohort development. These aspects are intended for better biospecimen characterization and pre-analytic sample quality, which is an absolute necessity for sample sharing via BBMRI-ERIC and other research collaborations. The LGDB is an entity that can help enhance bioresource availability and accessibility at an international level, thereby improving research relating to human health.

## APPENDIX A. SUPPLEMENTARY DATA

eMaterial 1. Medical institutions involved in donor recruitment for Genome Database of Latvian population

eMaterial 2. Translated informed consent of the Genome Database of the Latvian population

eMaterial 3. DNA isolation from whole blood by phenol/chloroform method used in Genome Database of Latvian Population

eMaterial 4. Categories of collected data on participants of Genome Database of Latvian population

eMaterial 5. Biochemical measurement information available on participants of Genome Database of Latvian population

eMaterial 6. Research areas of Genome Database of Latvian population sample application

## References

[1] Lugovska R, Vevere P, Andrusaite R, Newborn screening for PKU and congenital hypothyroidism in Latvia. Southeast Asian J Trop Med Public Health. 1999;30(Suppl 2):52–53. 11400783

[2] Marga M, Denisova A, Sochnev A, Two HLA DRB 1 alleles confer independent genetic susceptibility to Graves disease: relevance of cross-population studies. Am J Med Genet. 2001;102:188–191. 10.1002/ajmg.1431 11477614

[3] Nikitina Zake L, Cimdina I, Rumba I, Major histocompatibility complex class I chain related (MIC) A gene, TNFa microsatellite alleles and TNFB alleles in juvenile idiopathic arthritis patients from Latvia. Hum Immunol. 2002;63:418–423. 10.1016/S0198-8859(02)00385-3 11975986

[4] Tracevska T, Jansone I, Broka L, Mutations in the rpoB and katG genes leading to drug resistance in Mycobacterium tuberculosis in Latvia. J Clin Microbiol. 2002;40:3789–3792. 10.1128/JCM.40.10.3789-3792.2002 12354882 PMC130873

[5] Human Genome Research Law, of the Republic of Latvia. http://www.vvc.gov.lv/export/sites/default/docs/LRTA/Likumi/Human_Genome_Research_Law.doc. 2017. Accessed 02.02.17.

[6] Lee JH, Park Y, Choi JR, Comparisons of three automated systems for genomic DNA extraction in a clinical diagnostic laboratory. Yonsei Med J. 2010;51:104–110. 10.3349/ymj.2010.51.1.104 20046522 PMC2799962

[7] Spjuth O, Krestyaninova M, Hastings J, Harmonising and linking biomedical and clinical data across disparate data archives to enable integrative cross-biobank research. Eur J Hum Genet. 2016;24:521–528. 10.1038/ejhg.2015.165 26306643 PMC4929882

[8] Ērglis A, Dzērve V, Pahomova-Strautiņa J, A population-based cross-sectional study of cardiovascular risk factor in Latvia. Medicina (Kaunas). 2012;48:310–316. 22885365

[9] Latvian Population Census 2011. http://www.csb.gov.lv/en/statistikas-temas/population-census-30761.html. 2017. Accessed 02.02.17.

[10] Nelis M, Esko T, Mägi R, Genetic structure of Europeans: a view from the North-East. PLoS One. 2009;4:e5472. 10.1371/journal.pone.0005472 19424496 PMC2675054

[11] Bojesen SE, Pooley KA, Johnatty SE, Multiple independent variants at the TERT locus are associated with telomere length and risks of breast and ovarian cancer. Nat Genet. 2013;45(4):371–384, 384e1–2. 10.1038/ng.2566 23535731 PMC3670748

[12] Couch FJ, Wang X, McGuffog L, Genome-wide association study in BRCA1 mutation carriers identifies novel loci associated with breast and ovarian cancer risk. PLoS Genet. 2013;9:e1003212. 10.1371/journal.pgen.1003212 23544013 PMC3609646

[13] Sekar A, Bialas AR, de Rivera H, ; Schizophrenia Working Group of the Psychiatric Genomics Consortium Schizophrenia risk from complex variation of complement component 4. Nature. 2016;530:177–183. 10.1038/nature16549 26814963 PMC4752392

[14] Kalnina I, Kapa I, Pirags V, Association between a rare SNP in the second intron of human Agouti related protein gene and increased BMI. BMC Med Genet. 2009;10:63. 10.1186/1471-2350-10-63 19602223 PMC2714840

[15] Kalnina I, Zaharenko L, Vaivade I, Polymorphisms in FTO and near TMEM18 associate with type 2 diabetes and predispose to younger age at diagnosis of diabetes. Gene. 2013;527:462–468. 10.1016/j.gene.2013.06.079 23860325

[16] Peculis R, Lace B, Putnina A, HFE-related hemochromatosis risk mutations in Latvian population. Ann Hematol. 2015;94:343–344. 10.1007/s00277-014-2157-2 25015053

[17] Rovite V, Maurins U, Megnis K, Association of F11 polymorphism rs2289252 with deep vein thrombosis and related phenotypes in population of Latvia. Thromb Res. 2014;134:659–663. 10.1016/j.thromres.2014.07.011 25091233

[18] Rovite V, Petrovska R, Vaivade I, The role of common and rare MC4R variants and FTO polymorphisms in extreme form of obesity. Mol Biol Rep. 2014;41:1491–1500. 10.1007/s11033-013-2994-4 24385306

[19] Sällman Almén M, Rask-Andersen M, Jacobsson JA, Determination of the obesity-associated gene variants within the entire FTO gene by ultra-deep targeted sequencing in obese and lean children. Int J Obes (Lond). 2013;37:424–431. 10.1038/ijo.2012.57 22531089 PMC3595467

[20] Leja M, Shums Z, Nikitina-Zake L, Prevalence estimation of celiac disease in the general adult population of Latvia using serology and HLA genotyping. United European Gastroenterol J. 2015;3:190–199. 10.1177/2050640615569379 25922680 PMC4406903

[21] BBMRI-ERIC. http://www.bbmri-eric.eu/news-events/1177/. 2017. Accessed 02.02.17.

[22] Yin P, Lehmann R, Xu G Effects of pre-analytical processes on blood samples used in metabolomics studies. Anal Bioanal Chem. 2015;407:4879–4892. 10.1007/s00216-015-8565-x 25736245 PMC4471316

[23] Kamlage B, Maldonado SG, Bethan B, Quality markers addressing preanalytical variations of blood and plasma processing identified by broad and targeted metabolite profiling. Clin Chem. 2014;60:399–412. 10.1373/clinchem.2013.211979 24305685

[24] Common Minimum Technical Standards and Protocols for Biological Resource Centres dedicated to Cancer Research (IARC). http://ibb.iarc.fr/standards/index.php. 2007. Accessed 02.02.17. 33539055

[25] OECD Best Practice Guidelines for Biological Resource Centres. http://www.oecd.org/sti/biotech/oecdbestpracticeguidelinesforbiologicalresourcecentres.htm. 2007. Accessed 02.02.17.

[26] Rai AJ, Gelfand CA, Haywood BC, HUPO Plasma Proteome Project specimen collection and handling: towards the standardization of parameters for plasma proteome samples. Proteomics. 2005;5:3262–3277. 10.1002/pmic.200401245 16052621

[27] Hubel A, Spindler R, Skubitz AP Storage of human biospecimens: selection of the optimal storage temperature. Biopreserv Biobank. 2014;12:165–175. 10.1089/bio.2013.0084 24918763

[28] Elliott P, Peakman TC; UK Biobank The UK Biobank sample handling and storage protocol for the collection, processing and archiving of human blood and urine. Int J Epidemiol. 2008;37:234–244. 10.1093/ije/dym276 18381398

[29] Huppertz B, Michaela B, Macheiner T, Sargsyan K Biobank Graz: the hub for innovative biomedical research. Open J Bioresources. 2016;3:p.e3 10.5334/ojb.20

[30] Zander J, Bruegel M, Kleinhempel A, Effect of biobanking conditions on short-term stability of biomarkers in human serum and plasma. Clin Chem Lab Med. 2014;52:629–639. 10.1515/cclm-2013-0705 24327528

[31] Matharoo-Ball B, Thomson BJ Nottingham Health Science Biobank: a sustainable bioresource. Biopreserv Biobank. 2014;12:312–316. 10.1089/bio.2014.0056 25340939

[32] Sargsyan K, Macheiner T, Story P, Sustainability in biobanking: model of Biobank Graz. Biopreserv Biobank. 2015;13:410–420. 10.1089/bio.2015.0087 26697910

[33] Seiler CY, Eschbacher J, Bowser R, Sustainability in a hospital-based biobank and university-based DNA biorepository: strategic roadmaps. Biopreserv Biobank. 2015;13:401–409. 10.1089/bio.2015.0076 26697909 PMC4724783

[34] Burton PR, Hansell AL, Fortier I, Size matters: just how big is BIG?: Quantifying realistic sample size requirements for human genome epidemiology. Int J Epidemiol. 2009;38:263–273. 10.1093/ije/dyn147 18676414 PMC2639365

[35] Somiari SB, Somiari RI The future of biobanking: a conceptual look at how biobanks can respond to the growing human biospecimen needs of researchers. Adv Exp Med Biol. 2015;864:11–27. 10.1007/978-3-319-20579-3_2 26420610

[36] Lee YS Genetics of nonsyndromic obesity. Curr Opin Pediatr. 2013;25:666–673. 10.1097/MOP.0b013e3283658fba 24240285

[37] Zawati MH, Knoppers B, Thorogood A Population biobanking and international collaboration. Pathobiology 2014;81:276–285. 10.1159/000357527 25792216

[38] European Society of Human Genetics Data storage and DNA banking for biomedical research: technical, social and ethical issues. Eur J Hum Genet. 2003;11(Suppl 2):S8–S10. 10.1038/sj.ejhg.5201115 14718935

[39] Public Population Project in Genomics and Society. http://www.p3g.org/about-p3g. 2017. Accessed 02.02.17.

