# Supplementary material for: Genome Database of the Latvian Population (LGDB): Design, Goals, and Primary Results
Source: J Epidemiol. 2018 Aug 5;28(8):353–60. doi: 10.2188/jea.JE20170079 (PMC6048300; doi:10.2188/jea.JE20170079)
Supplement: Supplementary file 1 [file je-28-353-s001.pdf]

eMaterial 1. Medical institutions involved in donor recruitment for Genome Database of Latvian population

Pauls Stradins Clinical University Hospital
Oncology Centre of Latvia, Riga East Clinical University hospital
Clinic of medical genetics and prenatal diagnostics
Hospital “Linezers”, State hematology centre
Children`s Clinical University Hospital
In-patient Department “Gailezers”, Riga East Clinical University hospital
Riga City 1st hospital
In-patient Department “Bikernieki”, Riga East Clinical University hospital
Liepaja oncology hospital
Daugavpils oncology hospital
Riga Maternity hospital
LLC Clinic of Latvia dermatology institute
Latvian Centre of Infectious Diseases, Riga East Clinical University hospital
Centre of Tuberculosis and Lung Diseases
LLC Clinic “Piramid”
Latvia Stomatology institute
LLC Health Centre no. 4
Social care centre “Gailezers”
Social care centre “Raudas”
Riga Stradins University, Research laboratory of human molecular genetics
Daugavpils regional hospital
LLC health centre “Health”
LLC health centre “Therapy”
Jugla Medical centre
LLC health centre „Jaunkemeri“
LLC “Railway medical centre”
Centre of diabetes
LLC “Dzirciema health centre”
Medical centre “Elite”
Health centre of Jelgava
Dr. Maurina Vein Clinic
LLC „ORTO“ – clinic of orthopedy, sport traumatology and vertebra surgery
LLC health centre “Valeo”
Health centre “Ziepniekkalns”
Health centre “Plavnieki”
LLC health centre „AURA-R“
Clinic “Your doctor”
Doctor practice in endocrinology of Dr. I. Amoliņa, Dr. D. Teterovska, Dr. I. Lagzdina, Dr. N. Kanunnikova, Dr. M. Keisa, Dr. H. Dalecka
Family doctor practice of Dr. M. Pilenge, Dr. I. Andersone, Dr. I. Petrova, Dr. O. Golube, Dr. N. Zaharenkova, Dr. E. Satalova, Dr. I. Belevica, Dr. N. Rogoza, Dr. B. Berzina, Dr. A. Marcenoka, Dr. A. Cvetkova, Dr. I. Sviklane. Dr. Z. Torbejeva

eMaterial 2. Translated informed consent of the Genome Database of the Latvian population

Information about the Genome Database of the Latvian population

Dear Sir or Madam!
We invite you to participate in the project of the development of the Genome Database of Latvian population in collaboration with Latvian Biomedical Research and Study centre. We kindly ask you read the following information carefully before signing the consent form.

The aim of the project:
Scientists have discovered that almost all disease development is facilitated by the interaction between heredity and harmful environmental factors. The aim of the project is to collect information about the heredity and genes of the population of Latvia, and environmental factor which might affect disease development, and store this information in a united database. These genetic studies would allow us to obtain new information about disease development and make steps for prophylaxis to attain or prevent these diseases. During the development of the Genome Database of Latvian population several scientific groups are participating to study gene influence on disease development. These researches will receive anonymized samples of your biological material and information about your health status from the Genome Database of Latvian population.

Description of enrolment procedures:
A certified medical specialist will draw a sample of your venous blood. Afterwards, a trained interviewer will help you to fill in the health and hereditary questionnaire, in which you will be asked to answer questions about you and your relatives regarding hereditary and environmental factors, which might affect disease development. Your health and hereditary information, and blood samples will be coded to ensure confidentiality.
Coded blood samples will be transported to Latvian Biomedical Research and Study centre and processed, to make sure your genetic information could be suitable for genetic analysis and long term storage in specifically designed restricted authorization area. Only project participants and persons with specific permissions will be allowed to use this coded information for scientific research only. Your personal data and other information that is acquired during enrolment and research process will not be accessible, with exclusion of cases provided in the legal regulation of the Republic of Latvia, these examples are described further in this form.
The informed consent, sample and data identification code will be stored at State Genome Registry.

Duration of the project:
Genome Database of Latvian population is developed and maintained at Latvian Biomedical Research and Study centre for an unlimited period of time. Your consent form will be stored at State Genome Registry for 75 years after the last revisions made.

Potential risks:

The participation in genome research will not harm your health. You will be required to provide a blood sample the same way as in case of a regular blood test. In rare cases a local hemorrhage or in very rare cases local skin inflammation can develop at the site of venipuncture.

Data confidentiality and the rights of the project participant:

Confidentiality of your personal data, health and hereditary information, genetic research results are ensured by “Human Genome Research Law” and “Personal Data Protection Law” of the Republic of Latvia. This information will be stored in a restricted access area and will not be provided to your relatives, insurance companies, or employers. Obtained data and tissue materials will not be used for commercial purposes and will not be illegally given to third parties.

The data collected about you in Genome Database of Latvian population will be given to the requesting parties only in cases that are provided to State Genome Registry, in cases described in “Human Genome Research Law”, and to you personally based on a submitted written application. Information about your health and results of genetic research (not your personal information!) will be accessible to researchers, who’s projects are approved by the Genome Research council and the Central Medical Ethics committee. With your written consent (with exclusion of emergency medical help) your health information will be accessible for your doctor in case of reasonably grounded request.

You have rights to access your data that are stored at Genome Database of Latvian population and supplement new data. You also have rights to prohibit supplementation, renewal and control of your data stored at Genome Database of Latvian population or limit the extent of your genome research. You have rights at any moment to withdraw your participation in the genome research project, in this case your tissue material, health status and hereditary information will be destroyed in Genome Database of Latvian population and in State Genome Registry.

Voluntary participation:

Your participation is voluntary. Your refusal to participate in this project will not harm you by any means.

Potential benefits:

Participation in this project will not give you any immediate benefit, but will serve for the generation of novel knowledge about diseases and heritability, that in future might help you, your relatives or the general society. In accordance with “Human Genome Research Law” you do not have the rights to demand charge for your biomaterial, health and hereditary information, as well as for the use of the genetic research results.

Contact person:

If you have additional questions please contact Prof. Janis Klovins at Latvian Biomedical Research and Study centre, Ratsupites str. 1-1, Riga, LV – 1067, Latvia.

Consent document of the gene donor*
(in relation to Genome Database of Latvian population)

I. (filled in by gene donor or his/her legal guardian)

1. I have received and carefully studied the written information about the aim of the genetic research project, content, duration and potential risks. All my questions were answered promptly and understandably. I had enough time to consider my decision to become a gene donor.

2. I am informed that I have rights to access the data regarding genetic research of my biological sample. I understand that I do not have the right to demand any charge for my samples, my health or genealogy research, as well as the use of the research results.

3. Supplementation, renewal and control of my health status in the genome database is:

☐ allowed
☐ prohibited

4. Research of my genome is:

☐ not limited
☐ limited to a certain extent (provide limitations)

____

5. Abroad shipment of my biologic material and health status information for genetic research is:

☐ allowed
☐ prohibited

6. If during the research of my genome any information about potential threats to my health or the health of my relatives will be discovered, I:

☐ agree that this information is communicated to me
☐ agree, that this information is communicated to me in cases if the risk for the health is preventable
☐ do not want this information to be communicated to me

7. I agree to be a gene donor in the genome research project voluntary, without any charge. I agree that for the purposes of the genetic research my tissue samples will be taken and health status and (or) genealogy can be recorded. I understand that at any time I have the right to withdraw my participation without providing any explanations. In this case my tissue sample, health status and any personal information will be destroyed.

**Gene donor**	
Name and Surname<br>(in block letters)	
Personal ID No.	
Address	
Date<br>(day, month, year)	
Signature	

II

**Gene researcher/Doctor of the gene donor**	
Name and Surname<br>(in block letters)	
Employment	
Signature	
Date<br>(day, month, year)	
Place of consent completion	

*"gene donor" – term used in "Human Genome Research Law" of the Republic of Latvia to describe a participant consenting to the research of his/her genetic material. Therefore, "gene donor" is a historical term, however, overtime biobank has evolved having much broader sense of patient recruitment and biosample application.

eMaterial 3. DNA isolation from whole blood by phenol/chloroform method used in Genome Database of Latvian Population

Reagents and solutions

Stored at room temperature:

Cell Suspension solution: 25 mM EDTA, 200 mM NaCl

DNA Hydration solution: 1mM EDTA, 10 mM Tris-HCl pH 7,6

10% SDS (Sodium dodecil sulfate)

70 % Ethanol Solution

100 % Isopropanol

Chloroform

Stored at + 4°C:

RBC (red blood cell) lysis solution: 320 mM sucrose, 5 mM $MgCl_2$, 1% Triton-X-100, 10 mM Tris-HCl pH 7,6

Phenol pH 8 (buffered with 100 mM Tris-HCl, pH 8)

Stored at - 20°C:

Proteinase K (0.6 U/μl)

DNA isolation procedure

1. Purification of white blood cells

1) The whole blood is collected in a blood collection tube containing EDTA anticoagulant.
2) Blood sample in collection tube is centrifuged for 15 min at +4˚C and 4000 rpm (centrifuge must be cooled to +4˚C).
3) Plasma is removed by pipetting, leaving 0.5 – 1 cm plasma level over buffy coat and red blood cell fraction.
4) Carefully, with sterile cut-off tip, all buffy coat is removed, containing small portion of the plasma, but avoiding the transfer of blood cells from the red fraction, cells are transferred to empty 15 mL tube.

Note: If by accident buffy coat fraction is contaminated with red blood cell fraction, this tube can be repeatedly centrifuged for 15 min at +4˚C and 4000 rpm, and repeated buffy coat collection carried out.

5) The rest of the blood is discarded in biological waste.

2. Lysis of red blood cells

6) 10 mL of RBC Lysis solution is added to 15 mL centrifuge tube containing white blood cells and the tube inverted three times (without use of vortex and avoiding vigorous shaking).
7) Mixture is incubated for 15 min at +4˚C.
8) After incubation 15 mL tubes are centrifuged for 15 min at +4˚C and 4000 rpm.
9) The supernatant is removed by decanting to biologic waste container and the white blood cell pellet is kept.

10) The cells are washed with 5 mL Cell Suspension solution by inverting the tube (avoiding vigorous shaking or vortexing) and centrifuged for 5 min at +4˚C and 4000 rpm. The supernatant is removed.

3.Lysis of white blood cells

11) 5 mL of Cell Suspention solution is added to the pellet.
12) Tube is mixed on rotator until cells are suspended, no longer than 5 min.
13) 0.4 mL 10% SDS solution is added and mixed by inverting the tube 3 times.
14) 5 µl of Proteinase K is added and mixed by inverting the tube 3 times.
15) Mixture is incubated overnight at +50 ˚C, by mixing the tube several times at the start of incubation.

4. DNA isolation

16) 5 mL of phenol is added to lysed white blood cells and mixed on rotator for 15 min.
17) Mixture is centrifuged for 10 min at + 20 ˚C, 4000 rpm.
18) Upper fraction is transferred in new 15 mL tube, without touching phenol and remaining cell sediment.
19) 5mL chlorophorm is added to transferred fraction and mixed on rotator for 5 min.
20) Mixture is centrifuged for 10 min at +20 ˚C, 4000 rpm.
21) Upper fraction is transferred in new 15 mL tube, without touching chlorophorm and remaining cell sediment.

Note: If transferred fraction contains too many cell sediment, repeatedly 5mL chlorophorm can be added and mixed for 5 min on rotator, centrifuged for 10 min at 4000 rpm and upper fraction transferred in new 15mL tube.

22) Slowly, on the inner side of the tube, 5 mL isopropanol is added to transferred fraction and mixed by inverting tube several times (avoiding shaking), until DNA precipitates (appears medusa).

Note. In case if DNA precipitate do not form, sample with isopropanol is left overnight at -18 ˚C

23) Mixture is centrifuged for 10 min at +20 ˚C, 4000 rpm.
24) All supernatant is discarded and 5mL of 70% ethanol added, mixture is vortexed thoroughly and mixed on rotator for 2 min.
25) Mixture is centrifuged for 10 min at +20 ˚C, 4000 rpm.
26) The supernatant is carefully removed by decanting, making sure that sediment stays on walls of the tube. Tube is turned upside down and sediment dried for 10 min at room temperature or until it is completely dry.
27) 0,5 mL or 1 mL of DNA Hydration Solution is added to the sediment, depending on size of precipitated DNA, and tube placed on rotator on slow rate over the night.
28) Isolated DNA is stored for a week at +4 ˚C to completely dissolve. Total yield and quality of DNA is measured by NanoDrop 1000 Spectrophotometer.

eMaterial 4. Categories of collected data on participants of Genome Database of Latvian population

Group	Collection	Theme	Question or series of questions
Health and Hereditary questionnaire	Interview-based, self-reported	Sociodemographic data	Sex<br>Year of birth<br>Age<br>Place of birth<br>Nationality<br>Marital status<br>Sibs<br>Children<br>Level of education
		Participant health history	History of oncological diseases<br>Heart and cardiovascular diseases<br>Endocrine and chronic diseases
		Use of medicines	Regularly used medicines<br>Side-effects
		Lifestyle	Smoking history<br>Alcohol consumption<br>Physical exercises
		Allergies and symptoms	Allergies<br>Symptomatics
		Environmental factors	Radiation<br>Harmful environmental and workplace factors<br>Passive smoking
		Diet	Diet type<br>Products and beverages
		Family sociodemographic data	Place of birth<br>Nationality<br>Longevity
		Family socioeconomic status	Accommodation and living
		Family health history	History of oncological<br>Heart and cardiovascular<br>Endocrine and chronic diseases
Physical Measurements	Measured or self-reported	Anthropometric Measurements	Height<br>Weight<br>Hip circumference<br>Blood pressure<br>Heart rate
Medical diagnosis	Doctor's diagnosis	ICD-10 diagnosis codes	Diagnosis on the day of recruitment

eMaterial 5. Biochemical measurement information available on participants of Genome Database of Latvian population

Biochemical parameter	Number of LGDB participants	Unit of measurement
HDL-cholesterol	5894	mmol/l
LDL-cholesterol	5843	mmol/l
Triclycerides	5790	mmol/l
Cholesterol	5781	mmol/l
Glucose in plasma	3713	mmol/l
C reactive protein	942	mg/l
Fasting glucose	814	mmol/l
Glucose in plasma after 120 min	769	mmol/l
HbA1c	695	%
C peptide	657	ng/ml
Creatinine	589	µmol/l
Glomerular filtration rate	523	ml/min
ALAT	450	U/l
Non-HDL cholesterol	443	mmol/l
Insulin	355	µU/ml
Apolipoprotein A1	295	mg/dl
Homeostatic Model Assessment - Insulin Resistance	253	mmol/l
Apolipoprotein B	238	mg/dl
Creatine kinase	222	U/l
Vitamin B12	70	pg/ml
Total bilirubin	57	µmol/l
**Other biochemical parameters available for less than 50 LGDB participants**		
Blood tests: Complete blood count, free T4, thyroid stimulating hormone, antibodies to TSH receptor, ASAT, albumin, ASO, Anti-GAD-65, and other parameters		
Urine tests: albumin/creatine ratio, protein, pH, erythrocytes, nitrite, ketone bodies, glucose, bilirubin, urea, uric acid, creatinine and other parameters		

eMaterial 6. Research areas of Genome Database of Latvian population sample application

Research area	Research type	Participants (number in each study) Diagnosis (ICD-10 codes), inclusion criteria	Genes /Biological material	Publications
Arthritis	Genetic association studies of various autoimmune diseases in Latvian population	Cases (n=95/95/840) M05, M06, E10, L40, K50, K51, M45 patients<br><br>Controls (n=262/262/412) matched individuals without autoimmune diseases	*IRF5*, *MMEL1*, *CTLA4*, *REL*, *STAT4*, *TNFAIP3*, *IRF5*, *BLK*, *CD40*, *IL23R*, *PTPN22*, *IL10*, *ERAP1*, *TNFA*, *TRAF1*, *TGFB1*	https://www.ncbi.nlm.nih.gov/pubmed/22150086
Breast and ovarian cancer	Genetic studies on breast and ovarian cancer	Breast (n=26) and ovarian (n=21) cancer patients	*BRCA1*, *BRCA2*, genome wide association	https://www.ncbi.nlm.nih.gov/pubmed/27836010<br>https://www.ncbi.nlm.nih.gov/pubmed/27796716<br>https://www.ncbi.nlm.nih.gov/pubmed/27601076<br>https://www.ncbi.nlm.nih.gov/pubmed/27463617<br>https://www.ncbi.nlm.nih.gov/pubmed/27459855<br>https://www.ncbi.nlm.nih.gov/pubmed/27117709
Cancer immunology	Studies for identification and validation of antigens and biomarkers in cancer (gastric, prostate, thyroid) and research of anti-tumor resistance molecular and cellular mechanisms	Cases (n=487/228/221/10) C50, C34, C18, C43, C73, C16, C91, C16<br><br>Controls (n=274/413/186/10) matched cancer and autoimmune disease-free individuals	Autoantibodies against CT-antigens, tumour antigens, RNAseq of exosomal RNAs	http://www.ncbi.nlm.nih.gov/pubmed/21150711<br>http://www.ncbi.nlm.nih.gov/pubmed/19351608<br>http://www.ncbi.nlm.nih.gov/pubmed/22684876;<br>http://www.frontiersin.org/10.3389/conf.fimmu.2013.02.01082/event_abstract<br>http://www.ncbi.nlm.nih.gov/pubmed/25171478
Coronary heart disease	Association studies of common SNPs with coronary heart disease and related conditions	Cases (n=683/1781) I20.1, I42, I43, I21<br><br>Controls (n=826/1725) matched no heart related conditions	*ADORA3*, *P2Y1*	https://www.ncbi.nlm.nih.gov/pubmed/21675873<br>http://www.ncbi.nlm.nih.gov/pubmed/21643756
Essential tremor	Case-control studies of genetic factors potentially associated with essential tremor	Cases (n=141/104): patients with Essential Tremor (G25)<br><br>Controls	LINGO1 (10 SNPs listed in publication), 16 STR markers in *ETM1* and *ETM2* locus, coding sequences of *DRD3* gene	http://www.ncbi.nlm.nih.gov/pubmed/21741293<br>http://www.ncbi.nlm.nih.gov/pubmed/18637033

		(n=130/116): healthy individuals		
Familial hypercholesterolemia	Next-generation-sequencing-based identification of familial hypercholesterolemia-related mutations and association of common SNPs with lipid levels in a Latvian population	Individuals with increased blood lipid levels, without CHD (n=92/16/1273/706)	*APOB*, *LDLR*, *PCSK9* and *LDLRAP1*, listed in publications (144 SNPs in more than 30 locus)	http://www.ncbi.nlm.nih.gov/pubmed/26415676 https://www.degruyter.com/view/j/prolas.2015.69.issue-1-2/prolas-2014-0011/prolas-2014-0011.xml http://www.ncbi.nlm.nih.gov/pubmed/25606439 http://www.ncbi.nlm.nih.gov/pubmed/23675527
Genetics of rare disease	Genetic risk factors of non-syndromic cleft palate, cleft lip and inherited neuromuscular diseases	Cases (n=258/81/90) Individuals with non-syndromic cleft palate, cleft lip, limb-girdle muscular dystrophy, hyperCKemia, or cardiomyopathies<br><br>Controls (n=192/100/100) Individuals without non-syndromic cleft palate, cleft lip, limb-girdle muscular dystrophy, hyperCKemia, or cardiomyopathies	listed in publication (40 genes and 587 SNPs), *CAV3*	http://onlinelibrary.wiley.com/doi/10.1002/bdra.20791/abstract;jsessionid=1FADC2310463D1405FE2A8B724F14B7A.f01t03 http://onlinelibrary.wiley.com/doi/10.1002/bdra.20700/abstract https://www.ncbi.nlm.nih.gov/pubmed/27142102
Hemochromatosis	*HFE*-related hemochromatosis risk mutations in Latvian population	Cases (n=1805) random database participants	*HFE*	https://www.ncbi.nlm.nih.gov/pubmed/25015053
Melanoma	Genetic studies of malignant skin melanoma	Cases (n=200/243/137/477/243/20/17/47/125/96) C43, familial melanoma patients<br><br>Controls (n =200/-/-/255/-/-/-/-/-/-) matched without cancer	*MC1R, PARP1, MITF, VDBP*, *CDKN2A, CDK4*	https://www.ncbi.nlm.nih.gov/pubmed/23522749. http://onlinelibrary.wiley.com/doi/10.1002/ijc.28796/epdf http://www.ncbi.nlm.nih.gov/pmc/articles/PMC3490389/pdf/pcmr0025-0384.pdf http://www.sciencedirect.com/science/article/pii/S2210776215000423# http://www.ncbi.nlm.nih.gov/pmc/articles/PMC4065372/ http://www.ncbi.nlm.nih.gov/pmc/articles/PMC3607098/ http://www.ncbi.nlm.nih.gov/pubmed/?term=10.1097%2FCMR.0b013e3283287d3e http://www.ncbi.nlm.nih.gov/pubmed/?term=PMID%3A+17505264 https://www.ncbi.nlm.nih.gov/pubmed/23546221
Obesity	Association studies of common and rare SNPs with obesity and genome wide	Cases (n=1135/24/380/380) to various extent increased BMI	*AGRP*, *FTO*, *MC4R*, genome-wide analysis	https://www.ncbi.nlm.nih.gov/pubmed/19602223 https://www.ncbi.nlm.nih.gov/pubmed/25010727 https://www.ncbi.nlm.nih.gov/pubmed/22531089 http://www.ncbi.nlm.nih.gov/pubmed/24385306

	examination of DNA methylation markers in relation to aging and obesity	Controls (n=-/22/380/380) normal BMI		
Other research areas	Molecular and genetic research of anthropological and paleopathological samples and aspects of longevity	Paleopathological samples and age selected participants (n=772/121/20)	mtDNA analysis, telomere analysis	http://www.ncbi.nlm.nih.gov/pubmed/25660060<br>https://www.ncbi.nlm.nih.gov/pubmed/26411886
Pituitary adenoma	Genetic association studies of pituitary adenoma (acromegaly and other types)	Cases (n=48/143) E22.0, E22<br><br>Controls (n =571/354) matched individuals without E22.0, E22	*SSTR5*, *SSTR2*, *DRD2*, *MEN1*, *AIP*, *PRKAR1A*, *GNAS*	https://www.ncbi.nlm.nih.gov/pubmed/21810856<br>https://www.ncbi.nlm.nih.gov/pubmed/27185868
Schizophrenia	Genome wide association studies of schizophrenia	Cases (provided by researchers)<br><br>Controls from (n=100) matched individuals without schizophrenia	Genome wide association	https://www.ncbi.nlm.nih.gov/pubmed/26854805<br>https://www.ncbi.nlm.nih.gov/pubmed/26814963<br>https://www.ncbi.nlm.nih.gov/pubmed/26663532
Type 2 diabetes	MetfoGENE, OPTIMED studies, for genetic variation in metformin pharmacogenetic factors and pharmacokinetic of metformin; genetic association studies of type two diabetes and related complications	Cases (n=53/466/37/987/201) Longitudinal study E11patients with documented metformin use and side-effects; E11, E10<br><br>Controls (n=193/-/-/1080/125) match individuals without E11, E10	*SLC22A1*, *SLC22A2*, *SLC47A1*, Illumina VeraCode GoldenGate assay (52 genes, 192 SNPs listed in publication); *TCF7L2*, *TMEM18*, *FTO*, *GLO1*	http://www.ncbi.nlm.nih.gov/pubmed/22735389<br>https://www.ncbi.nlm.nih.gov/pubmed/23860325<br>https://www.ncbi.nlm.nih.gov/pubmed/22441719<br>https://www.ncbi.nlm.nih.gov/pubmed/23201419<br>https://www.ncbi.nlm.nih.gov/pubmed/27859023<br>https://www.ncbi.nlm.nih.gov/pubmed/27609360<br>https://www.ncbi.nlm.nih.gov/pubmed/27500523
Venous thrombosis	Genetic association of polymorphisms with deep vein thrombosis and related phenotypes	Cases (n=177) I80<br><br>Controls (n=235) matched individuals without venous diseases	*SELE*, *FGG*, *SERPINC1*, *GP6*, *CYP4V2*, *F11*	http://www.ncbi.nlm.nih.gov/pubmed/25091233
